# Secondary Spontaneous Pneumothorax in a COVID-19 Recovered Patient

**DOI:** 10.7759/cureus.16415

**Published:** 2021-07-16

**Authors:** Joseph Sahagun, Amit Chopra, Alan G David, David Dao, Subramanyam Chittivelu

**Affiliations:** 1 Pulmonary Medicine, University of Illinois College of Medicine-Peoria, Peoria, USA; 2 Pulmonary Medicine, University of Illinois College of Medicine-Peoria, Peoria , USA; 3 Pulmonary and Critical Care Medicine, University of Illinois College of Medicine at Peoria - Order of Saint Francis Medical Center, Peoria, USA

**Keywords:** covid-19, pneumonia, spontaneous pneumothorax, secondary pneumothorax, covid-19 pneumonia

## Abstract

Coronavirus disease 2019 (COVID-19) is an infectious disease primarily affecting the lungs with a spectrum of post-viral complications. There are well-described examples of pneumonia, empyema, pneumomediastinum, and spontaneous pneumothorax cases following COVID-19 infection within the literature. However, there is insufficient evidence implicating the cause of spontaneous pneumothorax in COVID-19 recovered patients. We present a previously infected COVID-19 patient who developed a secondary spontaneous pneumothorax two weeks after recovering. A review of the literature for similar cases was limited and therefore includes a summary of recommendations. Overall, the literature establishes that pneumothorax can occur during different phases of COVID-19 in patients without a history of pulmonary disease or barotrauma and is not necessarily associated with the severity of the viral infection. As in the case of our patient, the culmination of chronic inflammatory changes and an acute exacerbation from COVID-19 further predisposed him to a secondary spontaneous pneumothorax. In summary, all cases of recovered COVID-19 patients should maintain close follow-up with their physician and seek medical attention if acute respiratory symptoms develop.

## Introduction

Over the past year, coronavirus disease 2019 (COVID-19) has rapidly spread in the United States. In turn, pulmonary radiological findings from this disease have become well documented, including post-viral complications [1-3]. There are increased reports of spontaneous pneumothorax in patients within the literature, which were thought to be an uncommon sequela of the infection. However, the development of the complication is not well understood, with some sources citing the virus as the cause while others are implicating pneumonia, poor intubation technique, or barotrauma [4-7]. In cases without proof of barotrauma, a secondary spontaneous pneumothorax may develop in the context of COVID-19 in 1% of patients [8]. Nonetheless, whether spontaneous pneumothorax results from barotrauma, COVID-19, or post-viral pneumonia, it is imperative to distinguish the issue rather than implicate the virus to address any underlying systemic problems to improve mortality in COVID-19 patients. Herein, we report a case of a patient who developed a secondary spontaneous pneumothorax two weeks after recovering from COVID-19. Though the complication is uncommon, the prompt identification and management of spontaneous pneumothorax are essential to improve outcomes in COVID-19 patients.

## Case presentation

We present a 72-year-old male with multiple comorbidities such as hypertension, Parkinson's disease, dementia, hyperlipidemia, and previous chronic tobacco use at half pack per day for 20 years admitted with acute hypoxic respiratory failure secondary to post COVID-19 superimposed bacterial pneumonia requiring ICU placement. Of note, he had never been formally diagnosed with chronic obstructive pulmonary disease (COPD) or emphysema and never required home oxygen at baseline. The patient was a poor historian due to his dementia, so history was primarily provided by his caregiver and review of patient records. The patient was previously diagnosed with COVID-19 and eventually recovered and then managed at home with conservative over-the-counter treatment. However, over the four days preceding admission, he continued to spike periodic fevers prompting his caregiver to call for EMS. En route, his SpO_2_ ranged between 81% and 85% on room air, and after arrival, he was placed on a high-flow nasal cannula (HFNC). A portable chest x-ray was obtained in the ED, which demonstrated bibasilar opacities concerning pneumonia. In addition to pulmonary hygiene, he was treated with a standard course of antibiotics (a five-day course of azithromycin, ceftriaxone) and methylprednisone 20 mg IV for three days followed by prednisone acetate 10 mg tablets daily for three days.

**Figure 1:**
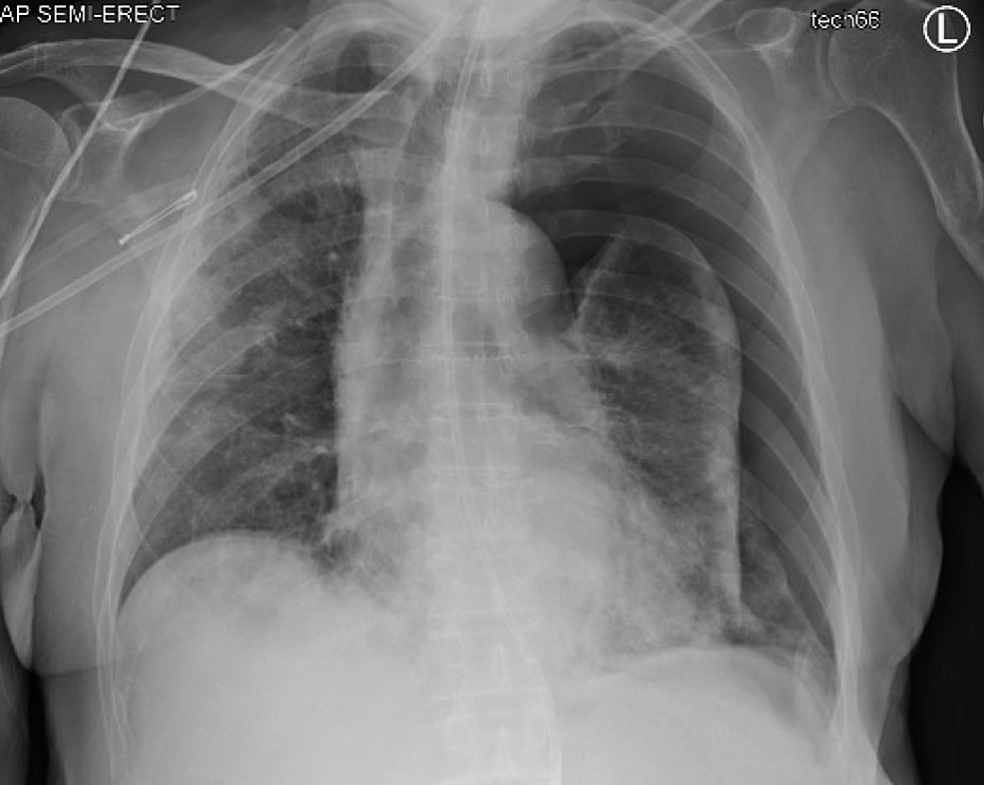
Left lung collapse with slight tracheal deviation to the right consistent with a secondary spontaneous pneumothorax

**Figure 2:**
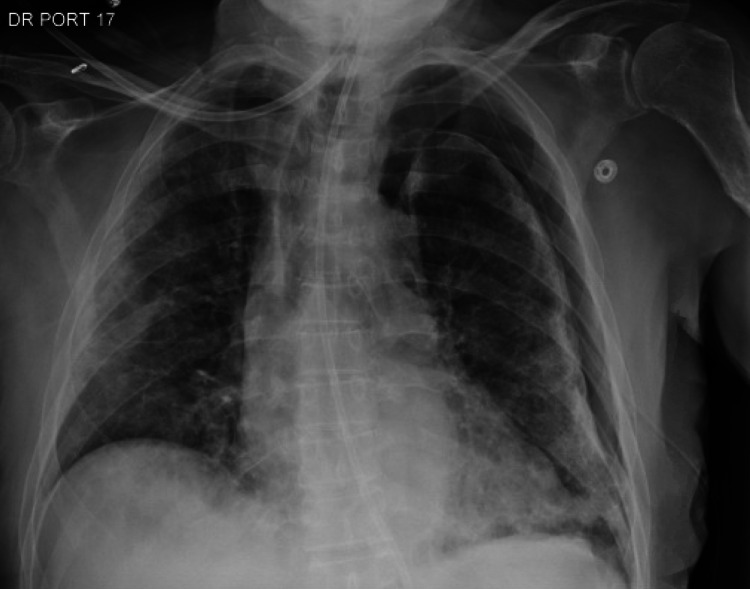
Left lung collapse with slight tracheal deviation to the right consistent with a secondary spontaneous pneumothorax

**Figure 3:**
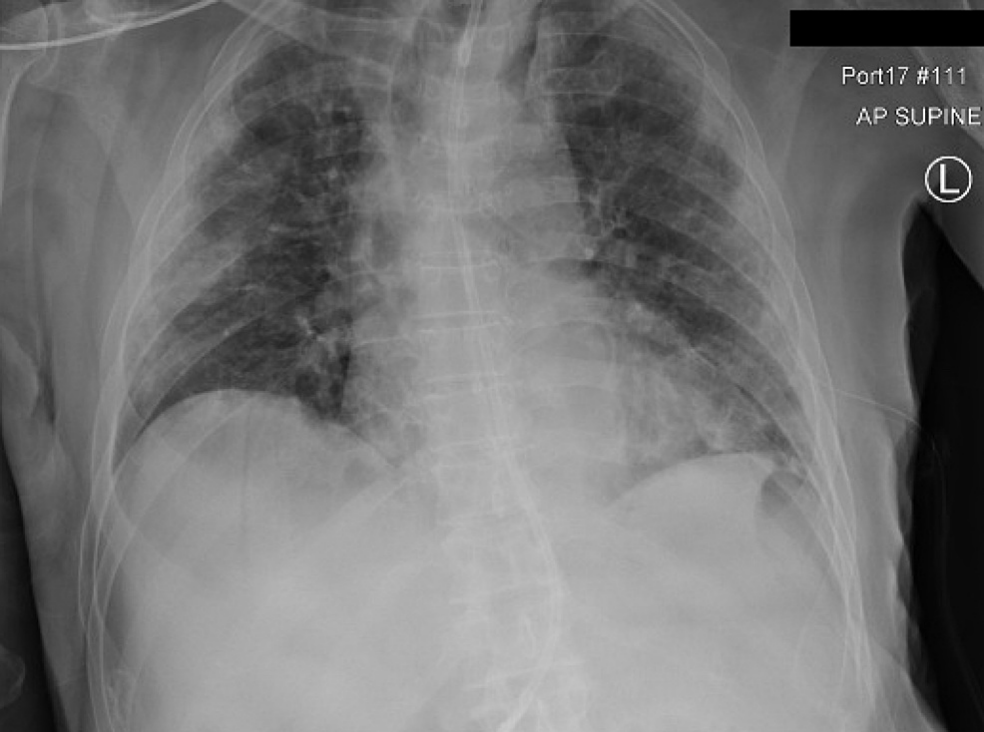
Status post chest tube removal demonstrating a resolved pneumothorax with new bilateral multifocal lung opacities concerning pneumonia

On day 8 of the hospital admission (day 17 from the initial COVID-19 diagnosis), the patient had significant oxygen desaturation below 80%. A subsequent chest x-ray (Figure 1) confirmed a sizable left-sided pneumothorax with partial collapse of the left lung and mediastinal shift. The inpatient pulmonary service was called for chest tube insertion and further management. A chest tube was placed at the bedside to continuous suction of -20 cm H_2_O with a repeat x-ray (Figure 2), indicating improved aeration. With supplemental oxygen provided via a nasal cannula, the patient’s saturation rose above 90%. The patient was also noted to have a robust non-productive cough, but it is unclear when the cough first developed or how long it had been present. On day 12, the chest tube was set to water seal, and the patient was continuously monitored. On day 13, the patient continued to be stable; hence, the pulmonary service decided to clamp the chest tube. A subsequent and final chest x-ray (Figure 3) did not show any pneumothorax, indicating resolution. The chest tube was thus removed on the morning of day 14. The radiology report in Figure 3 demonstrated persistent multifocal bilateral lung opacities concerning pneumonia. However, the actual likelihood of pneumonia was low since the patient lacked systemic symptoms such as fever and tachycardia and had a prior course of antibiotics during the hospital stay. It is possible that this could have been bilateral pleural effusions, although the possibility of pneumonia had to be considered.

During this hospitalization, the patient began developing significant dysphagia and was subsequently started on Keofeed tube feeds, but his power of attorney objected. Given his considerable medical comorbidities and dysphagia issues, goals of care meetings were held with his guardian and healthcare power of attorney, who elected to transition the patient to complete comfort care with a plan to discharge home with hospice.

## Discussion

The exact pathogenesis of pneumothorax in patients with COVID-19 has not been entirely elucidated, and a significant part of the difficulty stems from how infrequently it occurs since only 1% of all hospitalized COVID-19 patients develop this complication [9-12]. Within the literature, structural alterations in the lung parenchyma from COVID-19 may increase the likelihood of spontaneous pneumothorax. Notable changes include surface protein disruption, such as downregulation of surfactant, loss of extracellular matrix and basement membrane, and hypercoagulability due to significant NF-κB/NFKB2 pathway activation within COVID-19-infected lung tissue [1,7,9,11]. In part, these changes promote a hypoxic microenvironment where the tissue's capacity to recover is diminished and gradually becomes unable to withstand the intra-alveolar pressure culminating in a pneumothorax.

Another proposed mechanism entails pneumothorax secondary to pulmonary cyst rupture [13]. Pulmonary remodeling may develop before barotrauma from intubation or inflammatory processes such as COPD with emphysema, cystic fibrosis, tuberculosis, lung cancer, pneumocystis carinii pneumonia, lymphangioleiomyomatosis, Langerhans cell histiocytosis, and COVID-19 [14,15]. We also implicate the disruption of type II pneumocytes as predisposing one to spontaneous pneumothorax in COVID-19 since the virus enters cells through the angiotensin-converting enzyme-2, which is abundant in type II alveolar cells [16]. In addition to synthesizing surfactants, another critical role of type II pneumocytes is to mitigate damage by facilitating the regeneration of the alveolar epithelium following injury [17]. Disruption of these cells prevents tissue turnover and regeneration, which serves as a nidus for cysts and scars [2,18]. However, using a ventilator to manage the symptoms of this disease may be another reason underlying the formation of pulmonary cysts in patients affected by this virus [2]. Therefore, it is difficult to distinguish the exact cause of pneumothorax in an intubated patient with COVID-19.

Additionally, although rare, cough-induced pneumothorax should also be considered a contributing factor in our patient developing a spontaneous pneumothorax and pneumomediastinum [9,19]. The “Macklin effect” postulates that a large pressure gradient generated against a closed glottis can result in alveolar rupture, potentially leading to spontaneous pneumothorax, pneumomediastinum, and subcutaneous emphysema [20]. Coughing, sneezing, vomiting, and Valsalva maneuver have been associated with spontaneous pneumomediastinum and pneumothorax via the Macklin effect [19]. In the absence of obvious barotrauma, cough-induced spontaneous pneumothorax should be considered a possible etiology.

Our patient was admitted due to acute hypoxic respiratory failure secondary to COVID-19 pneumonia with superimposed bacterial infection, treated with azithromycin and ceftriaxone, but unexpectedly developed a spontaneous pneumothorax late in his hospital stay. From a review of his medical records, a previous CT report from 2014 described mild scarring around the lingular segment of the left lobe along with small pulmonary nodules and bullae. Although the patient was never formally diagnosed with emphysema or COPD, these findings suggest the patient had an increased risk of developing a secondary spontaneous pneumothorax even before his COVID-19 infection. Ultimately, cytokine and cell-mediated damage to type II alveolar cells from his COVID-19 infection in conjunction with the patient’s smoking history and potential barotrauma from HFNC likely led to the formation of pleural blebs which could have easily ruptured from excessive coughing according to the Macklin effect, thereby explaining the patient’s pneumothorax.

## Conclusions

This case highlights a significant clinical scenario of late occurring spontaneous pneumothorax accompanying COVID-19 pneumonia in a person with no history of chest trauma or mechanical ventilation. Clinicians overseeing the management of such patients should be aware that pneumothorax can develop due to complications from SARS-CoV-2, which, contrary to most of the previously documented cases, may even occur at the tail-end of their hospitalization. In this case, the patient was a prior smoker who had a non-productive cough before developing the pneumothorax. Throughout the hospital course, he was subjected to HFNC, which contributed to pneumothorax as a form of barotrauma. We ultimately hypothesize that this patient’s pneumothorax could be due to a culmination of acute inflammatory changes induced by COVID-19 on top of his already damaged lungs, further worsening the condition of the lung parenchyma forming pleural blebs that were easily ruptured by the patient’s coughing and exposure to HFNC. In conclusion, a thorough review of the patient's history, pulmonary risk factors, disease progression, and physical exam findings combined with radiological imaging can help identify a secondary spontaneous pneumothorax within COVID-19 patients.
